# Supplementary material for: Mitochondrial translation failure represses cholesterol gene expression via Pyk2-Gsk3β-Srebp2 axis
Source: Life Sci Alliance. 2024 May 8;7(7):e202302423. doi: 10.26508/lsa.202302423 (PMC11079605; doi:10.26508/lsa.202302423)
Supplement: Supplementary file 10 [file LSA-2023-02423_TableS1.docx]

**Table S1.** Primers used for PCR in this study

| Primer | Sequence | |
| --- | --- | --- |
|  | F | R |
| *Srebp2* | CCATCTTCCCCTCTCTTTCC | AGGGAAGATCCTGGGAGAAA |
| *Acat2* | TGAATTAAAGGCATGCACCA | TCAGCCTGGAAGAGGTCACT |
| *Hmgcs1* | TTTGATGCAGCTGTTTGAGG | CCACCTGTAGGTCTGGCATT |
| *Hmgcr* | GGTGTTCAAGGAGCATGCAA | TTGGTGCACGTTCCTTGAAG |
| *Mvk* | TGGCCTGCACCTTCTAGACT | GGTGGTTCCTTAGGGGACAT |
| *Mvd* | AAGCAGACGGGCAGTACAGT | CCTGGAGGTGTCATTGAGGT |
| *Pmvk* | GAAGATTGTGGAAGGCGTGT | TCTGACTCAGCATCGTCCAC |
| *Idi1* | CCTCAAAACCCAGAGATCCA | ACTCCTTCCCACTTCCACCT |
| *Fdps* | TCCAGGTCCAGGACGACGACTAC | CGCCTCATACAGTGCTTTCA |
| *Sqle* | CCTGTTGGGTTGCTTTCAAT | CACGTGGACTCCCTTTCAAT |
| *Lss* | GCTGCATGTGGTGTATGGAC | GAGAAACGTGCTCCTGGAAG |
| *18s* | CGCGGTTCTATTTTGTTGGT | AGTCGGCATCGTTTATGGTC |
| *Srebp1* | GATCAAAGAGGAGCCAGTGC | TAGATGGTGGCTGCTGAGTG |
| *Fdft1* | TCGGAGGAAAGCAAGACTGT | TTTGTTCCTGCGTGTCGTAG |
| *Fbw7* | GTGATAGAGCCCCAGTTCCA | TCCATGGGCTGTGTATGAAA |
| *hACAT2* | GCAGGTGTTCCTTCAATGGT | CACAGCTTTTAGGCCTGACC |
| *hSREBP2* | TGGCTTCTCTCCCTACTCCA | GAGAGGCACAGGAAGGTGAG |
| *hHMGCS1* | CAAAAAGATCCATGCCCAGT | AAAGGCTTCCAGGCCACTAT |
| *hHMGCR* | GTCATTCCAGCCAAGGTTGT | GGGACCACTTGCTTCCATTA |
| *hMVK* | GCTCAAGTTCCCAGAGATCG | ATGGTGCTGGTTCATGTCAA |
| *hMVD* | AGGACAGCAACCAGTTCCAC | GTGTCGTCCAGGGTGAAGAT |
| *hPMVK* | CGGAGAGTGTCTGACATCCA | AAGTTGTCCAGGCCACATTC |
| *hFDPS* | AGGGCAATGTGGATCTTGTC | GAAAGAACTCCCCCATCTCC |
| *hIDI1* | GGCTGAAAGCTGAGCTAGGA | ATTTCACCACTGGCTGCTTT |
| *hSQLE* | GTCTCCGGAAAGCAGCTATG | AAAAGCCCATCTGCAACAAC |
| *hLSS* | TTCCTGAGGCTCTCACAGGT | CCCTCCATCTGGATTTCTCA |
| *hNSE* | GTCCCACGTGTCTTCCACTT | TGGGATCTACAGCCACATGA |
| *hTH* | GCTCAGGAGCTATGCCTCAC | ACCTAGCCAATGGCACTCAG |
| *h18S* | AAACGGCTACCACATCCAAG | CCTCCAATGGATCCTCGTTA |

The sizes of PCR products ranged from 150 to 250 nucleotides in length. F, forward primer; R, reverse primer.
